# Protein structure modelling and evaluation based on a 4-distance description of side-chain interactions

**DOI:** 10.1186/1471-2105-11-374

**Published:** 2010-07-12

**Authors:** Vladimir Potapov, Mati Cohen, Yuval Inbar, Gideon Schreiber

**Affiliations:** 1 Department of Biological Chemistry, Weizmann Institute of Science, Rehovot, Israel

## Abstract

**Background:**

Accurate evaluation and modelling of residue-residue interactions within and between proteins is a key aspect of computational structure prediction including homology modelling, protein-protein docking, refinement of low-resolution structures, and computational protein design.

**Results:**

Here we introduce a method for accurate protein structure modelling and evaluation based on a novel 4-distance description of residue-residue interaction geometry. Statistical 4-distance preferences were extracted from high-resolution protein structures and were used as a basis for a knowledge-based potential, called Hunter. We demonstrate that 4-distance description of side chain interactions can be used reliably to discriminate the native structure from a set of decoys. Hunter ranked the native structure as the top one in 217 out of 220 high-resolution decoy sets, in 25 out of 28 "*Decoys 'R' Us*" decoy sets and in 24 out of 27 high-resolution CASP7/8 decoy sets. The same concept was applied to side chain modelling in protein structures. On a set of very high-resolution protein structures the average RMSD was 1.47 Å for all residues and 0.73 Å for buried residues, which is in the range of attainable accuracy for a model. Finally, we show that Hunter performs as good or better than other top methods in homology modelling based on results from the CASP7 experiment. The supporting web site http://bioinfo.weizmann.ac.il/hunter/ was developed to enable the use of Hunter and for visualization and interactive exploration of 4-distance distributions.

**Conclusions:**

Our results suggest that Hunter can be used as a tool for evaluation and for accurate modelling of residue-residue interactions in protein structures. The same methodology is applicable to other areas involving high-resolution modelling of biomolecules.

## Background

Accurately predicting the protein structure is one of the major goals of computational structural biology. In the recent years, computational methods for evaluation and modelling of proteins and their interactions have emerged and become, in some areas, a viable alternative for time and resource-consuming structural biology experiments. However, predicting the structure of a protein from its amino acid sequence is still a very challenging task and new approaches to model and evaluate protein structures are needed.

All proteins share the same backbone, with their structure and function determined solely by the side chains of the 20 different amino acids. Therefore, a precise modelling of residue-residue and residue-backbone interactions is a crucial aspect in computational evaluation of proteins. Computational methods rely on a potential function to evaluate the interactions within proteins [1]. In general, two types of the potential functions currently exist: physics-based [2-4] and knowledge-based [5]. The former rely on the basic physical principles to describe the forces that drive structure and function of proteins. However, physical force fields applied to proteins rely on estimations and simplifications to make them computationally feasible [6]. Knowledge-based potentials (KBP) derive statistical preferences on different features from structural and sequence databases and implicitly capture the many factors affecting the protein in its natural environment. A few problems are related to statistical potentials including the Boltzmann assumption [7], additivity of terms [8], choosing a reference state [9] and transferability of potentials [10,11]. However, knowledge-based potentials have numerous applications and were successfully used in threading [12], validation of experimentally determined protein structures [13,14], *ab initio *structure prediction [15,16], decoy discrimination [17-19] and more.

**Figure 1:**
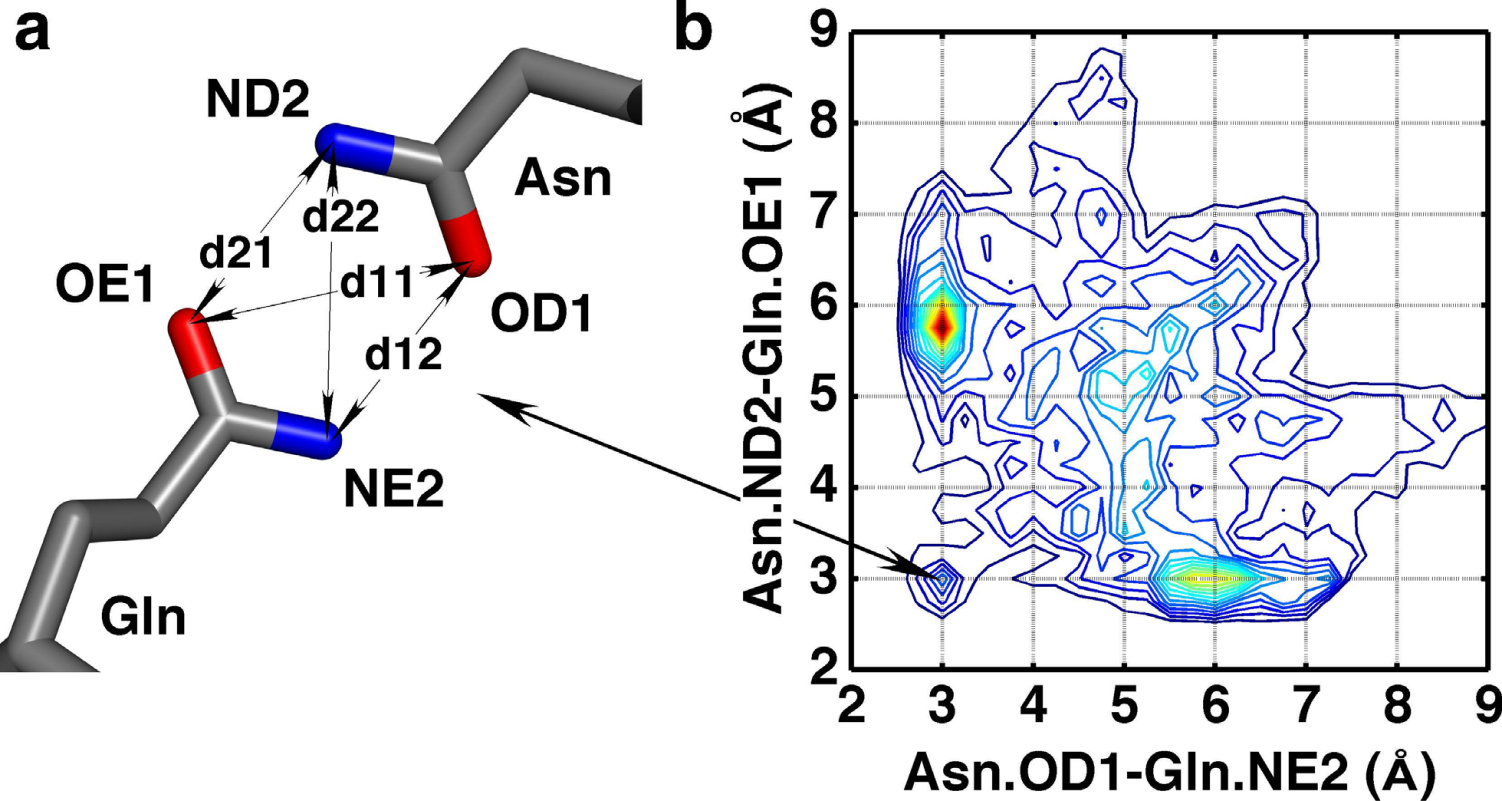
**Four-distance definition of inter-residue interactions for the Asn-Gln pair**. (a) To define the interaction, one pair of side chain atom is chosen in the first residue (Asn) and another pair is chosen in the second residue (Gln). The four distances (OE1-OD1, OE1-ND2, NE2-OD1, NE2-ND2) define mutual positions of chosen side chain atoms. (b) A 2-dimensional projection (OD1-NE2 versus ND2-OE1 distance) of the 4-distance distributions for the Asn-Gln pair. Peaks in the histogram indicate preferred distance combinations. The peak indicated by the arrow corresponds to the arrangement of side chain atoms in the panel (a). The histogram was built with a bin size of 0.25 Å, without smoothing. The histogram is represented as a contour plot generated with MATLAB. All 4-distance distributions can be viewed in the supporting web site http://bioinfo.weizmann.ac.il/hunter/.

In spite of the successful use of knowledge-based potentials in many areas, they have limited applicability for high-resolution description of side chain packing. In coarse-grained potentials, various approximations are used to avoid explicit consideration of side chains; thus, a residue can be represented as a single pseudo atom located at the center of mass of side chain atoms [20,21]. Such approximation limits the ability of coarse-grained potentials to reconstruct the fine details of residue-residue interactions. All-atom potentials treat side chain atoms explicitly and usually derive statistics on pairwise atom-atom distances [18,22]. However, each atom is considered separately and not as a part of a side chain. Therefore, information on mutual constraints between atoms is lost. Importance of orientation dependence of side chain packing was recognized and applied to fold recognition [23,24] and to analysis of pi-pi, pi-cation, and hydrophobic interactions [25]. In the recent study, orientation dependence was tackled by treating residues as being composed of rigid blocks [17]. Obtaining statistics on mutual orientation of the blocks allowed successful decoy discrimination and accurate side chain modelling [17,26]. An alternative approach for a detailed description of residue-residue interactions was recently presented by us [27]. This approach defines the interaction of two residues in terms of four distances between two pairs of atoms (Figure 1). The pairs of atoms are either chosen from the residues side chains or backbones so as to define side chain-side chain (*ScSc*) interactions or side chain-main chain (*ScMc*) interactions, respectively. Such description allows detailed analysis of preferable geometry of residue interactions [27].

In the current study, we utilise these 4-distance data to develop a novel knowledge-based potential, called Hunter. The statistical preferences on the geometry of residue-residue interaction were derived from a large set of high-resolution protein structures and normalized according to a random model. We demonstrate that Hunter can be successfully applied to evaluate and model protein structures. Examples include the discrimination of native structures from decoys, predicting side chain conformations in protein structures and homology modelling.

## Results

### Development of the knowledge-based potential

We began by defining pairs of atoms, among which four distances were calculated, for each of 190 *ScSc *and 18 *ScMc *residue-residue interactions. As described in the Methods, pairs of atoms with maximal number of contacts in 9394 high-resolution protein structures were identified (Atom Set 1; see Additional file 1: Table S1 and S2). More than 3 × 10^6 ^contacts were collected, with an average of 16,000 contacts per residue pair. As an alternative approach, we picked pairs of atoms manually based on consideration of functional group characteristics for a given amino acid (Atom Set 2). For example, carboxyl oxygens were chosen in Asp and Glu pairs, carbonyl oxygen and side chain nitrogen - in Asn and Gln pairs, terminal guanidyl nitrogens - in Arg, etc. (Table S3). Even though the number of contacts was similar in both cases, the set of atoms defined with our first approach demonstrated slightly better performance (Table 1), and was therefore used in the subsequent study. Throughout Hunter's development stages, we evaluated Hunter's performance by its ability to remodel side chains of known protein structures and calculated the RMSD between the model and the X-ray structure. The assumption being that the accuracy of side chain modelling is directly related to the overall performance of the KBP.

**Table 1:** Performance of the ScSc knowledge-based term in modelling

Atom set	Histogram type	Bin size, Å	Side chain RMSD, Å	
			
			Reduced RL	Full RL
Atom Set 1	Smoothed	0.25	2.37	2.95
		0.50	2.28	2.89
		0.75	2.41	3.00
		1.00	2.47	3.04
				
	Non-smoothed	0.25	2.69	3.32
		0.50	2.58	3.21
		0.75	2.55	3.08
		1.00	2.46	3.04
				
Atom Set 2	Smoothed	0.25	2.42	3.11
		0.50	2.38	3.03
		0.75	2.43	3.16
		1.00	2.53	3.17
				
	Non-smoothed	0.25	2.67	3.47
		0.50	2.62	3.27
		0.75	2.53	3.14
		1.00	2.43	3.06

To determine the best combination of parameters for the ScSc term, a set of 30 proteins was remodelled using the ScSc term only with different atom sets, bin sizes, and with or without histogram smoothing. The side chain RMSD was calculated for side chains atoms excluding Cβ atoms. Results for both the reduced and full rotamer libraries are presented.

**Figure 2:**
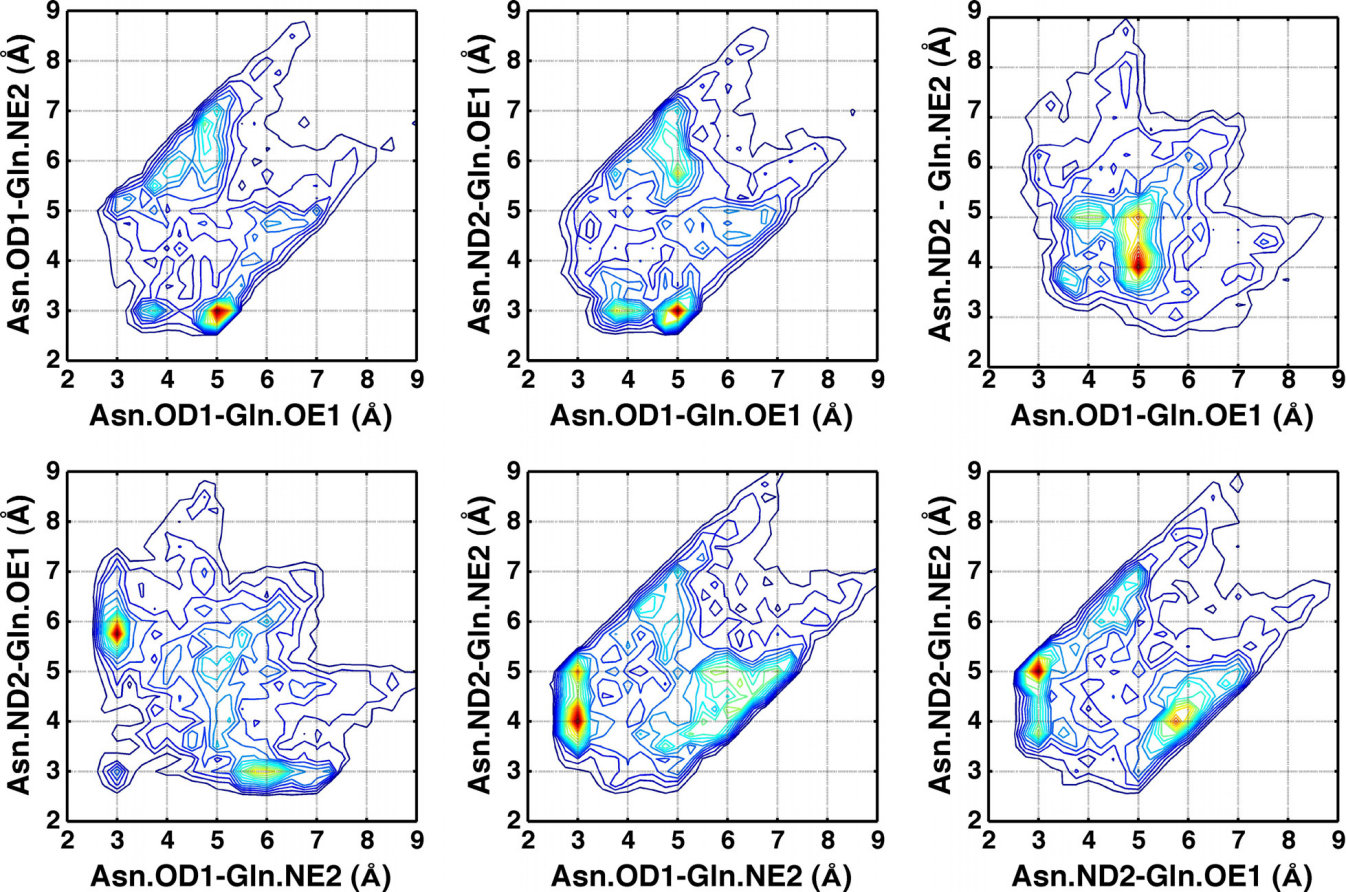
**Six 2-dimensional projections of the 4-distance distribution for the Asn-Gln pair**. Interaction between Asn and Gln is defined by four distances between OD1, ND2 atoms of Asn and OE1, NE2 atoms of Gln. The 4-distance data for Asn-Gln were collected from high-resolution protein structures (see Methods for details) and are displayed in six 2-dimensional projections. Each projection represents mutual distribution of two particular distances. The peaks in distributions indicate preferred mutual arrangements of atoms under consideration. The histograms were built with a bin size of 0.25 Å without smoothing. The histograms are represented as contour plots generated with MATLAB.

Given the atom contact pair, a residue-residue contact is represented by four distances {*dist*} = {*d*_11_,*d*_12_,*d*_21_,*d*_22_}, where *d*_*ij *_is the distance between atom *i *of the first residue and atom *j *of the second residue (Figure 1). All contacts collected for a given residue pair provide information regarding the preferred 4-distance combinations and the probability to observe them in native protein structures. To incorporate the derived distance information into the KBP, contact data collected for a particular residue pair were used to build a 4-dimensional histogram (Figure 2). For a given residue-residue contact (*AA*), each bin in the histogram gives the probability *P*({*dist*}|*AA*) for a defined set of distances. Such probability distributions were used to score interactions between any pair of residues by applying inverse Boltzmann relation (Equation 2).

The bin size greatly affects the histograms: using 1 Å interval per bin resulted in blurring the exact details of residue-residue interaction, however, using excessively small bins raised the level of noise because of the decreasing quanta of data points per bin. We tested bins of size 0.25 Å, 0.5 Å, 0.75 Å and 1 Å, and came to the conclusion that 0.5 Å best served the aims of this study. Considering the rapid growth of the PDB database, smaller bin definitions may be of advantage in the future. Still, even 0.5 Å interval per bin does not produce a smooth distribution. The small amount of data results in a high level of noise. Therefore, an additional step of smoothing was applied such that the bin value was recalculated as an average of values in adjacent bins and the bin itself. The different bin sizes and atom identities were tested for their accuracy to remodel the side chains of a set of 30 proteins (Table 1). As can be seen, the best performance was achieved using Atom Set 1, a bin size of 0.5 Å, and data smoothing. Interestingly, the reduced rotamer library performed much better than the full library, suggesting that the information content in the *ScSc *term is not sufficiently large to handle the additional degrees of freedom given in the full library (Table 1).

It should be noted that, in principle, there are various ways to chose pairs of atoms defining *ScSc *interactions. In addition to the two described above, we examined also the possibility to choose atom pairs that would maximize Kullback-Leibler distance (*D*_*KL*_): a "distance" from a real to a random distribution in our case. This would allow deriving the most informative set of atoms for a given set of PDB structures. Nevertheless, the performance of the *D*_*KL*_-set was worse than the two described above (data not shown). Among the described atom sets the preference was given to Atom Set 1 as it allows collecting better contact statistics, which is the main limiting factor in the 4-distance description. This set performed also slightly better, though, the difference between the two sets might be not very compelling (Table 1).

We found that smoothing improved performance of the *ScSc *term even though the current algorithm has the obvious drawback that the central bin value is overwhelmed by its neighbours. Nevertheless, the performance of the smoothed *ScSc *term was superior over non-smoothed one for the majority of bin sizes (Table 1). The same trend is observed for both Atom Set 1 and 2. It is interesting to note that the accuracy of non-smoothed *ScSc *term increases with a larger bin size, while with the smooth term an opposite trend is observed. Though non-smoothed distributions are more detailed, the current limitations in computational optimization of side chain conformations do not allow adequate sampling of such distributions (see discussion).

When using only the *E*_*ScSc *_term an RMSD of 2.28 Å was obtained for the reduced rotamer library and 2.89 Å for the full library (Table 1). As there are many other factors effecting side chain conformations in protein structures that are not covered by the *E*_*ScSc *_term, we added additional terms to Hunter: a *ScMc *contact term, a rotamer probability term and a modified Lennard-Jones term (Equation 1). Our knowledge-based potential was optimized on a set of 30 proteins and tested on a larger set of 94 protein structures. We noted that the combination of *E*_*rot *_and *E*_*lj *_terms by itself already gave reasonable accuracy in side chain modelling. However, adding the *E*_*ScSc *_term and the *E*_*ScMc *_term further increased the accuracy. Thus, using all four terms for modelling with full rotamer library gave 1.47 Å RMSD on a set of 94 structures (Table 2). Modelling with the reduced rotamer library resulted in 1.52 Å RMSD on the same set of structures. We found that the *E*_*ScMc *_term by itself did not perform well though combining it with *E*_*ScSc *_term gave improvement in side chain packing (Table 2). Two other terms were tested with the KBP, a solvation term (both the Lazaridis-Karplus approach [28] and the Eisenberg-McLachlan approach were tested [29,30]) and an entropy term based on the move acceptance ratio at each position during Monte Carlo minimization. Unfortunately, both terms did not improve side chain modelling accuracy and therefore were not included in the final potential (data not shown).

**Table 2:** Contribution of different terms to side chain prediction accuracy

	Side chain RMSD, Å		
	All	Buried	Exposed
E_ScSc_, E_ScMc_, E_rot_, E_lj_	1.47	0.73	1.72
E_rot_, E_lj_	1.52	0.77	1.78
E_ScSc_, E_ScMc_	2.28	1.39	2.61
E_ScSc_, E_rot_	1.87	1.36	2.05
E_ScSc_, E_lj_	2.04	0.97	2.41
E_lj_	2.13	1.04	2.51
E_rot_	2.25	2.05	2.29

The contribution of the different term to Hunter was evaluated by rebuilding side chain conformations on a test set of 94 proteins. The full rotamer library was used with Hunter (see Methods for details).

### Recognition of native protein structures

We investigated whether the *ScSc *term can be used to identify the native structure within a set of decoys. This would tell us about general applicability of the developed term for evaluating accuracy of residue-residue interactions in protein structures. We tested the *ScSc *term for its ability to identify native protein structure within the *Decoys 'R' Us *decoy sets [31]. Out of 34 decoy sets, the native structure was ranked first in 29 (Table 3). In the remaining 5 cases, two native structures were determined by NMR. The failure of Hunter in these cases may not be surprising, as NMR data do not comply with our 4-distance distributions [27]. For the other three cases the failure may be attributed to the low quality of the native structures (2.8 Å). Next, we compared Hunter's performance to four other methods [17,32]. We found that these methods performed quite similarly to ours: Hunter - 29/34, OPUS-PSP [17]- 31/34, DOPE [32]- 28/32, DFIRE [19]- 27/32 (see Table S4 for details). Most of the decoys in the *Decoys 'R' Us *sets differ significantly from the native structures (the closest structure had RMSD of ~4 Å). Therefore, the performance of Hunter was tested on additional high-resolution decoy sets [33], where the Cα RMSD (between decoys and native structures) ranges from 1.1 Å to 5 Å. Hunter correctly identified the native structure in 217 out 220 decoy sets. Again, for NMR structures the success rate was much lower, only 30 out of 80 (data not shown).

**Table 3:** Discrimination of native structures in *Decoys 'R' Us *multiple decoys sets

	Resolution, Å	Rank of native structure	Z-score of native structure	Number of decoys	RMSD range, Å
***4state_reduced***					
1sn3	1.20	1	5.3	660	1.3 - 9.1
4rxn	1.20	1	5.7	677	1.4 - 8.1
4pti	1.50	1	4.4	686	1.4 - 9.3
1ctf	1.70	1	4.7	630	1.3 - 9.1
1r69	2.00	1	6.6	676	0.9 - 8.3
3icb	2.30	15	2.5	654	0.9 - 9.4
2cro	2.35	1	4.1	673	0.8 - 8.3
***fisa***					
4icb	1.60	1	6.6	500	4.8 - 14.1
2cro	2.35	1	5.4	501	4.3 - 12.6
1fc2	2.80	33	1.6	501	3.1 - 10.6
1hdd-C	2.80	1	5.3	501	2.8 - 12.9
***fisa_casp3***					
smd3	?	1	7.1	1200	8.5 - 17.0
1bg8-A	2.20	1	6.1	1200	6.0 - 15.8
1bl0	2.30	1	6.0	972	3.6 - 18.2
1eh2	NMR	1	4.4	2413	4.0 - 15.3
1jwe	NMR	1	8.3	1407	7.8 - 20.9
***lattice_ssfit***					
4icb	1.60	1	5.1	1988	4.7 - 12.9
1ctf	1.70	1	6.2	1999	5.4 - 12.8
1fca	1.80	1	4.2	1986	5.1 - 11.4
1pgb	1.92	1	6.2	1997	5.8 - 12.9
1beo	2.20	1	6.6	1998	7.0 - 15.6
1dkt-A	2.90	1	5.5	1995	6.7 - 14.0
1nkl	NMR	1	5.2	1995	5.3 - 13.6
1trl-A	NMR	1	5.8	1998	5.4 - 12.5
***lmds***					
1igd	1.10	1	8.1	501	3.1 - 12.6
2ovo	1.50	1	5.6	348	4.4 - 13.4
4pti	1.50	1	5.2	344	4.9 - 13.2
1ctf	1.70	1	7.6	496	3.6 - 12.5
1b0n-B	1.90	1	5.3	498	2.4 - 6.0
1shf-A	1.90	1	7.7	437	4.4 - 12.3
2cro	2.35	1	7.7	501	3.9 - 13.5
1fc2	2.80	49	1.5	501	4.0 - 8.4
1bba	NMR	501	-3.8	501	2.8 - 8.9
1dtk	NMR	3	3.6	216	4.3 - 12.6

Each decoy structure and the native structure were scored and ranked using the Hunter's ScSc term only. Rank of 1 corresponds to the structure with the lowest score. The Z-score of the native structure was calculated to estimate Hunter's ability to discriminate the native structure from decoy structures. Resolution is of the X-ray structure.

**Figure 3:**
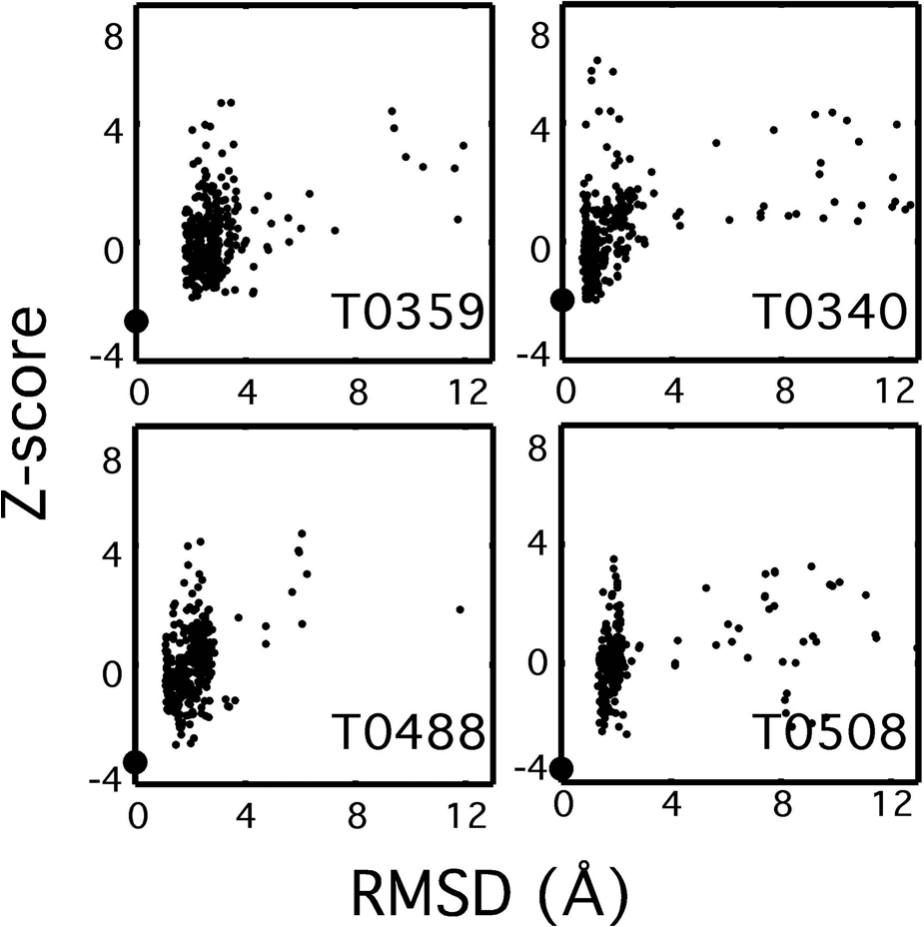
**Discriminating the native structures in four CASP 7/8 decoy sets (T0359, T0340, T0488, T0508) using Hunter**. Each structure in a particular decoy set was scored with Hunter *ScSc *term *only*. All obtained scores were converted to Z-scores. The Z-score of each decoy protein was plotted against its Cα-RMSD to the native structure. The native structure (0 Å RMSD) is shown as large circle, and has the lowest score (indicated as ranks in Tables S4 and S5). In most cases no funnel-like shape is observed.

A more challenging decoy set is to use CASP submissions. These represent decoys that other experts found compelling enough to submit for CASP. The *ScSc *term *only *was tested to recognize the native structure among a large set of modelled structures submitted for each of 73 targets solved by X-ray for high-accuracy template-based modelling in CASP7/8 (Table 4). Our *ScSc *term correctly identifies the native structure in 53 cases (73%). Considering only high-resolution targets (resolution < 2 Å), the *ScSc *term scores the target structure with rank 1 in 24 cases and rank 2 in two cases out of 27 targets. This suggests a significant difference in the detailed architecture of the modelled structures relative to the native structure. We found that among other tested methods, OPUS-PSP performed slightly better than Hunter while the other methods showed much lower discriminative power (Table S5). Figure 3 shows four examples of calculated Hunter scores for decoys taken from the CASP7/8 set. In all four cases the Z-score of the wild-type structures is the lowest. It is worth noting that no correlation is observed between RMSD and score of decoy structures and/or a funnel-like shape, suggesting a qualitative difference between the real structure and the models.

**Table 4:** Discrimination of native structures in CASP 7/8 decoys sets

	Resolution, Å	Rank of native structure	Number of decoys	Z-score of native structure	RMSD range, Å
***CASP7***					
T0288	1.1	2	373	2.3	1.6 - 11.2
T0359	1.4	1	383	2.7	1.8 - 12.8
T0340	1.5	1	416	2.0	0.7 - 8.9
T0324_D1	1.5	1	342	3.4	1.6 - 13.3
T0324_D2	1.5	1	400	3.2	1.2 - 10.7
T0305	1.6	1	321	2.3	0.9 - 19.8
T0332	1.6	1	343	2.9	1.5 - 10.9
T0366	1.7	1	414	2.7	0.9 - 8.0
T0291	1.8	1	252	1.9	2.0 - 19.2
T0290	1.8	2	196	2.3	0.5 - 13.4
T0313	1.9	1	366	2.9	2.4 - 19.0
T0311	1.9	1	403	2.8	1.4 - 11.1
T0295_D1	1.9	1	295	2.2	1.1 - 15.1
T0295_D2	1.9	1	235	2.2	1.1 - 23.9
T0303_D1	1.9	1	194	3.9	1.7 - 14.1
T0308	2.0	1	385	2.1	1.3 - 13.9
T0317	2.0	1	341	3.1	1.6 - 13.1
T0346	2.0	6	348	1.8	0.4 - 13.6
T0339_D2	2.1	1	399	2.8	1.7 - 15.7
T0345	2.1	1	314	2.2	0.8 - 16.0
T0367	2.2	1	337	3.3	2.0 - 17.2
T0292_D1	2.2	4	298	2.2	1.3 - 10.8
T0292_D2	2.2	1	174	4.3	3.0 - 13.8
T0315	2.2	1	384	2.8	0.9 - 13.0
T0334	2.5	1	328	2.0	1.5 - 45.5
T0326	2.5	1	281	2.2	3.1 - 29.3
T0328	2.8	1	317	2.9	1.7 - 40.1
***CASP8***					
T0488-D1	1.3	1	341	3.3	1.1 - 5.6
T0508-D1	1.5	1	283	3.5	1.3 - 11.8
T0459-D1	1.7	1	296	3.5	1.4 - 8.4
T0423-D1	1.7	1	349	3.2	1.2 - 15.1
T0454-D1	1.8	22	464	1.5	0.8 - 6.2
T0504-D3	1.8	1	241	3.8	1.2 - 21.6
T0445-D1	1.8	1	174	6.1	1.4 - 11.2
T0392-D1	1.8	1	327	2.2	1.2 - 8.1
T0447-D1	1.9	1	116	4.3	1.3 - 32.5
T0505-D1	1.9	1	246	3.0	1.3 - 10.3
T0506-D1	1.9	1	297	3.9	1.4 - 11.3
T0432-D1	1.9	1	169	3.9	1.4 - 17.1
T0388-D1	2.0	1	133	3.5	1.1 - 10.7
T0402-D1	2.0	1	310	4.2	1.5 - 22.6
T0418-D1	2.0	1	335	3.4	1.1 - 11.2
T0418-D2	2.0	1	344	4.2	1.5 - 10.2
T0422-D2	2.0	1	299	3.1	1.6 - 12.3
T0491-D1	2.0	12	319	2.3	1.6 - 10.9
T0428-D1	2.0	3	335	2.4	0.7 - 9.4
T0426-D1	2.1	3	168	2.7	0.5 - 6.0
T0396-D1	2.1	1	403	2.8	1.4 - 13.9
T0398-D1	2.1	1	273	2.5	0.6 - 33.5
T0398-D2	2.1	1	301	2.9	0.6 - 10.6
T0453-D1	2.1	3	322	3.2	1.3 - 8.0
T0435-D1	2.2	1	296	3.4	1.8 - 12.6
T0400-D1	2.2	1	270	3.9	1.3 - 11.3
T0452-D1	2.2	1	279	4.5	1.7 - 13.2
T0452-D2	2.2	1	319	3.2	1.0 - 15.0
T0486-D1	2.3	1	302	4.2	1.3 - 9.9
T0404-D1	2.4	1	317	3.7	0.9 - 7.4
T0479-D1	2.4	4	317	2.7	1.2 - 7.7
T0456-D2	2.5	2	331	2.6	2.7 - 8.8
T0390-D1	2.7	1	287	2.5	1.4 - 16.7
T0455-D1	2.7	1	297	3.3	1.4 - 8.3
T0416-D1	2.7	1	265	2.6	1.4 - 42.1
T0450-D1	2.7	3	263	2.6	1.5 - 53.5
T0458-D1	2.7	7	342	1.9	0.6 - 9.3
T0438-D1	2.8	5	254	2.8	1.3 - 11.2
T0438-D2	2.8	11	311	1.7	1.0 - 12.5
T0442-D1	2.8	11	250	1.4	1.1 - 25.1
T0442-D2	2.8	66	315	0.8	0.7 - 21.6
T0444-D1	2.8	7	314	2.2	0.9 - 7.8
T0461-D1	2.8	1	307	2.9	1.6 - 10.0
T0441-D2	2.9	1	271	3.1	1.8 - 7.2
T0470-D1	2.9	36	334	1.3	1.7 - 11.0
T0470-D2	2.9	16	324	1.6	1.0 - 13.4

See a legend of Table 3 for details.

### Monte Carlo side chain optimization

**Figure 4:**
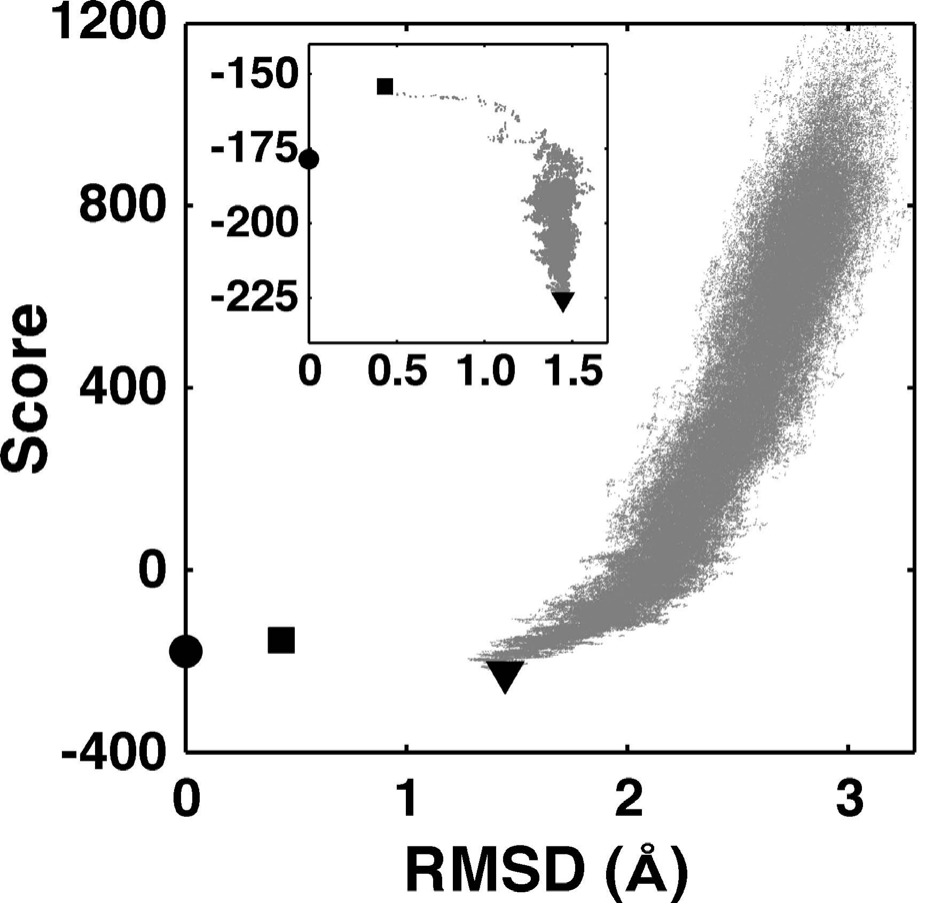
**Monte Carlo side chain optimization of *barnase-barstar *complex using Hunter**. A model of the complex (PDB ID 1BRS) with the lowest score has a side chain RMSD of 1.44 Å (filled triangle; gray dots in the plot are the conformations sampled during a MCSA run). The native structure (filled circle) is never attainable in the side chain modelling due to use of a discrete rotamer library. Instead, the best rotameric structure for the complex would have a RMSD of 0.43 Å (filled square). None of the conformations in the region between 0.43 Å and 1.44 Å is ever sampled. To investigate this problem, a MCSA run was started from the best-rotameric structure (see inset). As can be seen in the inset, such a MCSA run, nevertheless, converges to the same region as a standard run.

For side chain modelling the *ScSc *term was combined with the *ScMc*, the Lennard-Jones and the rotamer terms. Simultaneous optimization of a large number of side chain conformations is a computationally demanding task. In the current study, we used Monte Carlo Simulated Annealing minimization (MCSA). As a stochastic algorithm MCSA does not guarantee to identify a global minimum (GM). However, it is known to identify near-GM solutions efficiently [34]. We investigated the implementation of this minimization protocol and our potential for side chain modelling. During the side chain optimization of *barnase-barstar *complex (Figure 4), the model with the lowest score has an RMSD of 1.44 Å, while the native structure rebuilt using discrete rotamers (best-rotameric) has an RMSD of 0.43 Å. Due to rotameric constraints of the side chain optimization, the native structure is never attainable. Irrespectively of the rotamer library, MCSA never sampled the region between the best-rotameric structure and the structure with the lowest score. Starting the MCSA from the best-rotameric structure did not change the outcome (Figure 4 inset), indicating that the lowest scoring structure is dictated by our potential rather than by the implementation of the minimization protocol. While the best-rotameric structure is very close to the native one in terms of RMSD, its score is much higher. It might be that all structures with RMSD between 0.43 Å and 1.44 Å have a bad score because of the clashes resulting from the use of discrete rotamers. To further investigate this issue, a modified version of MCSA was implemented, where at each step one dihedral angle was modified arbitrarily by 5° for off-rotamer sampling. However, this did not improve significantly the results.

### Side chain prediction within proteins

We tested Hunter's performance for side chain placement on the set of 100 proteins compiled by *Word et al*. [35]. This set includes very high-resolution protein structures (1.7 Å or better, R-value of 20% or better, and sequence identity < 30%) with various minor corrections, including 180° flips of side chain amides (where needed). We excluded from our analysis six structures (PDB IDs: 1ETM, 1NOT, 1MET, 1CNR, 1EDM, 2ERL) since their chain length is less 50 amino acids, and these structures are mostly peptide-like. The side chains in 94 high-resolution protein structures were remodelled using the optimal combination of weights, and the modelled structures were compared to the native ones resulting in an average RMSD of 1.47 Å (Table 5; see also Table S6 for additional details). The RMSD for buried and exposed side chains was 0.73 Å and 1.72 Å. The χ_1_-angles were predicted correctly within ± 15° cutoff for 79%, 89%, and 72% of all, buried, and exposed residues respectively (59%, 75%, 51% when considering χ_1_- and χ_2_-angles). Even better results were obtained when using a subset of 49 protein structures, which are not part of a larger macromolecular assembly and do not contain ligands: 1.43 Å for all residues and 0.68 Å for buried ones (Table S6). For most of this study we used RMSD as a measure of side chain accuracy, as we believe that it better represents the accurate position of side chain atoms.

**Table 5:** Comparing performance of side modelling methods

Method	RMSD (Å)			Contact score	**χ**_**1 **_**(± 15°)**	**χ**_**1+2 **_**(± 15°)**
				
	All	Buried	Exposed		%	%
Hunter	1.47	0.73	1.72	39	79	60
OPUS-Rota [26]	1.56	0.91	1.80	35	77	56
SCAP [50]	1.72	1.00	1.96	37	69	46
SCCOMP [42]	1.72	1.03	1.96	34	69	49
SCWRL4.0 [51]	1.65	0.87	1.93	35	76	55

Side chains were rebuilt using five different methods in a set of 94 high-resolution protein structures. The average statistics on side chain prediction accuracy was calculated in terms of side chain RMSD, contact score, and chi-angle prediction accuracy. Side chain RMSD was calculated for side chain atoms except Cβ. A residue was considered as exposed or buried if its relative solvent exposed area is greater or less than 15%, respectively. Contact score reflects amount of correctly predicted atom-atom contacts and is calculated as described in Methods.

Comparing Hunter to other commonly used side chain placement methods (Table 5) shows that Hunter (RMSD = 1.47 Å) performed better on the same set of 94 proteins than SCWRL4 (1.65 Å), SCCOMP (1.72 Å), SCAP (1.72 Å) and OPUS-Rota (1.56 Å). Hunter is the slowest of these methods, with an average run time of 1 minute per protein versus 5 seconds for SCWRL and OPUS-Rota. However, the slowest component in Hunter is the Lennard-Jones term, while the *ScSc *and the *ScMc *terms are not computationally demanding. Interestingly, we noticed that prediction accuracy depends on the individual protein structure: modelling 94 proteins with different methods showed large differences between the proteins, independent of the method used. One common term that could explain this is the percentage of exposed residues in a protein: a correlation factor of 0.5 was found between per protein side chain RMSD and the percentage of exposed residues.

**Figure 5:**
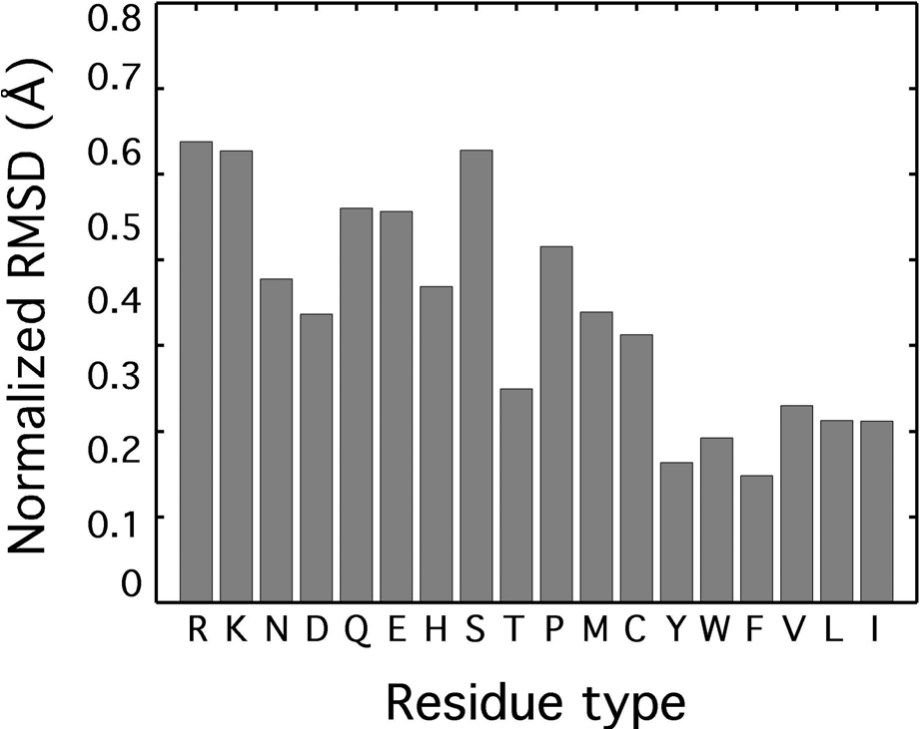
**Per residue side chain modelling accuracy**. Per residue RMSDs after modelling using Hunter were collected for all side chain conformations on the set of 94 models. Each per residue RMSD was normalized (see Table S7 for details), and an average normalized RMSD was calculated. Hydrophobic residues are on the right side of the plot while polar ones are on the left side.

**Figure 6:**
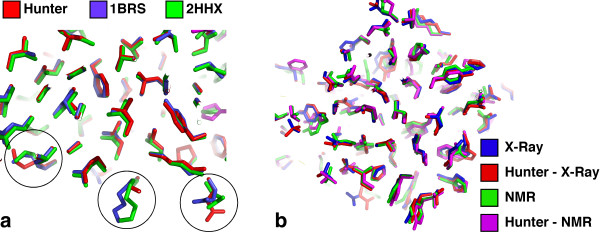
**Evaluating side chain prediction accuracy using Hunter in X-ray and NMR structures**. (a) Side chain conformations of the *barstar *structure were modelled using Hunter and compared to those determined in two different X-ray crystal structures. For most buried side chains, conformation predicted with Hunter is in a good agreement with observed conformations in the crystal structures. For a number of exposed residues (circled in the figure) Hunter's side chain conformation is different from those observed in crystal structures; however, their conformations differ also between the structures. (b) Side chains were rebuilt with Hunter starting from the X-ray or NMR structures of *barnase *(PDB IDs 1A2P and 1FW7, respectively; backbone RMSD between X-ray and NMR structure is 1.07 Å). RMSD between X-ray and NMR - 1.45 Å; X-ray modelled versus NMR modelled - 1.49 Å; X-ray versus NMR modelled - 1.54 Å; X-ray modelled versus NMR, 1.52 Å; X-ray versus X-ray modelled, 0.85 Å; NMR versus NMR modelled - 1.15 Å.

To compare the accuracy of modelling individual amino acids we used nRMSD (see Table S7 for details), which is the relation between the average RMSD of randomly placing a side chain and the RMSD for the modelled side chain (nRMSD of 1 Å is for random placement). Not surprisingly, hydrophobic and aromatic residues were modelled better than polar and charged residues as they tend to be more buried (Figure 5). Figure 6a provides a visual illustration of the accuracy of side chain modelling for the protein *barstar *(PBD ID 1BRS and 2HXX). Nearly all the buried side chains were placed in their correct conformation within the limit of the rotamer library, with a few exposed side chains adopting a wrong conformation. However, these surface exposed residues adopt different side chain conformations also in the two crystal structures of *barstar*, suggesting that the accuracy of modelling is inherently limited. In Figure 6b, we demonstrate modelled side chain conformations of *barnase *starting from the X-ray and NMR structures (PDB IDs 1A2P and 1FW7, respectively; backbone RMSD between X-ray and NMR structure is 1.07 Å). We show that Hunter's side chain prediction accuracy is within the difference between the X-ray and NMR structures, indicating that Hunter provides high-resolution information.

We have shown previously that the differences between interfaces and monomeric proteins relate to the amino acid composition but not to the chemistry of the interactions [36]. Therefore, we evaluated also the suitability of Hunter for interface remodelling. We identified 20 non-redundant protein-protein hetero-complexes solved to high resolution [37] and remodelled the side chains. In this case, the interface residues were modelled at the same level of accuracy as all residues (1.63 Å interface residues RMSD versus 1.67 Å all residues RMSD; Table S8). The overall worse RMSD values for the 20 protein-protein complexes comparing to the 94 monomeric proteins used above may be due to the lower resolution of the structures of the complexes (1.2-2.3 Å for the 20 protein-protein complexes versus 0.8-1.7 Å for the 94 monomers). Next, we modelled a set of 20 dimers which were solved to high resolution (similar to the monomer set of 94 structures), and indeed the average RMSD for Hunter improved to 1.57 Å.

### Side chain prediction in comparative modelling

So far, we used the native backbone to model side chains. While this is of academic interest, the real test would be to model side chains on homology-modelled structures [38]. Using templates always results in backbone inaccuracies in the final models that in turn affect the accuracy of side chain prediction. We collected CASP7 predictions submitted by the six top-performing groups for each target domain in the high accuracy template-based modelling category (HA/TBM) [39]. Then, we compared the submitted results to those generated by Hunter. As can be seen in Table 6, Hunter performed as good or better than the best side chain prediction method per model. We also remodelled the side chains of NMR structures, and found that the RMSD of the models equals that of the original NMR structures in relation with the equivalent X-ray structure (see also Figure 6b).

**Table 6:** Hunter's performance for side chain predictions in homology modelling of structures from CASP7

	Number of targets	RMSD, Å	Contact score	**χ**_**1 **_**(± 15°) %**	**χ**_**1+2 **_**(± 15°) %**
Hunter	28	**4.1**	**17.7**	**63**	**41**
TS004		4.1	17.1	60	37
					
Hunter	26	**3.7**	**20.9**	**61**	**40**
TS020		3.7	18.9	60	38
					
Hunter	27	**4.0**	**19.2**	**62**	**41**
TS186		4.0	16.0	60	37
					
Hunter	5	**3.0**	**27.6**	**69**	**46**
TS191		3.1	20.8	64	39
					
Hunter	6	**3.8**	**18.5**	**61**	**37**
TS397		3.8	16.5	59	38
					
Hunter	6	**3.9**	**18.0**	**63**	**39**
TS556		4.0	17.8	60	38

The results of the top six groups in CASP7 (according to *Read and Chavali *[39]) were taken for examining Hunter side chain modelling accuracy in homology modelling. Best models submitted by each group for every target were collected and the average side chain prediction accuracy was determined (side chain RMSD, contact score, chi-angle prediction accuracy). Then, Hunter was used to rebuild side chains on the set same set of submissions, and side chain prediction accuracy was evaluated and presented below. For each set of submissions two rows are given. The first row shows side chain modelling accuracy with Hunter while taking backbone coordinates from the best model submitted by the corresponding group for each target. The second row shows performance in side chain modelling by the group in CASP7. The group name is given here according to the CASP7 experiment.

## Discussion

Here, we introduce Hunter, a novel structure modelling method. The method relies on a detailed description of residue-residue interaction geometry as derived from high-resolution protein structures. We demonstrate that such 4-distance description can be used successfully to evaluate protein structures and model side chain interactions with high accuracy. It should be noted that it would take at least 6 parameters to have a truly complete description of *ScSc *geometry. However, because of the limited size of the PDB, we opted to use four distances, which provides a good compromise between the number of parameters and the number of available data points. Other authors used three parameters, as for example in the classical treatment by Singh and Thornton to map the position of single atoms relative to a predefined part of the side chain [40]. We have also tried an alternative definition with three distances but found the results less satisfactory. In this study, we used a culled list of protein structures with a resolution of 2 Å or better and a maximum sequence identity of 90%. The relatively relaxed threshold on sequence identity did not adversely influence the derived statistics as even small changes in protein structures result in variation of constrained distances, thus the collected data is not redundant. In spite of the large number of protein structures in the database, for some residue pairs the number of extracted contacts was low, which led to a low bin count in the histograms. A large bin size may solve this problem; however, in such situation the distance constraints become blurred. Instead, smoothing was applied and the bin size of 0.5 Å was found to be optimal (Table 1). We found that even the simplest smoothing algorithm improves side chain prediction accuracy. This relates to several aspects of the 4-distance definition: one is the noise in distributions due to the limited amount of contact data, and the second is the mutual correlations in the 4-distance term. This results in rugged histograms, which in turn affects the efficiency of computational minimization. Smoothing allows lowering barriers and improving MCSA performance. Other computational methods like the dead-end elimination algorithm [41] might be beneficial with 4-distance distributions and are being currently studied. Another way to deal with histogram ruggedness is to represent probability density as a continuous function. To this end we tried the Gaussian Mixture Model, however, no improvement was achieved. A possible way to overcome limited 4-distance data is by hierarchical splitting of the histograms depending on population density. However, as this would slow the run time we did not implement this method.

Hunter was able to successfully identify native structures in multiple decoys sets. While decoys are similar to native structure, the details of residue-residue interactions are often incorrect. This is not the case for many of the protein structures determined by NMR, where the native NMR structure is already not optimal according to the KBP and therefore does not differ much from decoy structures. Similarly, we find that the resolution of the X-ray structure correlates with decoy prediction accuracy (Table 4). In the low-resolution structures the energy minimization has a significant contribution, suggesting that the accuracy of current minimization methods is limited [6,27]. In decoy recognition, Hunter is basically used as a "scoring" device, when geometry of the interaction is evaluated according to derived statistical preferences. In this case the ruggedness of histograms does not have a significant impact. Indeed, we found that smoothed and non-smoothed distributions could be used equally well for discriminating the native structure (data not shown). At the same time, the smoothing is crucial for side chain modelling, which involves sampling rugged histograms with discrete rotamers. Importantly, combining the *ScSc *term and Lennard-Jones term for discriminating the native structure within decoys sets does not improve the results in CASP7/8 sets. Presumably, the packing in the submitted models was optimized using the Lennard-Jones term while the 4-distance description is orthogonal to the commonly used terms. Thus, it probably has considerable and novel merit as an after-the-fact evaluation of predicted models (as done in the CASP tests).

Independently, the 4-distance *ScSc *and *ScMc *terms are insufficient to accurately model inter-residue interactions. While the *E*_*ScSc *_and *E*_*ScMc *_terms accurately define the mutual position of the constrained atoms, these terms do not define conformation of the entire side chain. Therefore, to penalize unfavourable side chain conformations, the *E*_*rot *_term was introduced. The *E*_*lj *_term was incorporated to optimize packing of the protein. Combining the four terms *E*_*ScSc*_, *E*_*ScMc*_, *E*_*rot *_and *E*_*lj *_produced the best results for modelling side chain conformations (Table 2). We note that while adding the *E*_*ScMc *_term to *E*_*ScSc *_alone improved the results, only a small improvement was observed when the *E*_*ScMc *_term was added to the combination of *E*_*ScSc*_, *E*_*rot *_and *E*_*lj *_terms (data not shown). We think that a partial overlap of the components in our KBP is the most plausible explanation for this fact. Presumably, *E*_*ScMc *_adds only limited information over what is already counted by *E*_*ScSc*_, *E*_*rot *_and *E*_*lj *_terms. We also tested a solvation term, and it did not improve side chain modelling accuracy. We thought of two possible explanations: it is possible that the KBP already implicitly captures solvation effects, taking into account that the protein is surrounded by water in the crystal environment. On the other hand, solvation term might be of less importance in side chain modelling contrary to protein design, as the amino acid sequence is fixed.

Hunter was effective for side chain modelling and allows modelling of different types of residue-residue contacts, such as hydrogen bonding and pi-cation interaction without explicitly introducing their geometric features. In this sense, it is similar to OPUS-PSP. However, while in Hunter the interaction between a pair of residues is defined in terms of 4 distances between only two pairs of atoms, OPUS-PSP decomposes side chains into rigid blocks and derives statistics on their preferred mutual orientation. While both methods performed equally well in discriminating native structure within high-resolution *Decoys 'R' Us *and CASP 7/8 multiple decoys, Hunter achieves better side chain prediction accuracy. We speculate that too many constraints (as in OPUS-Rota by considering interactions of individual blocks) may be counter productive in side chain modelling.

In modelling high-resolution structures, the RMSD values obtained by Hunter for buried, surface and all residues are 0.73 Å, 1.72 Å, 1.47 Å for the 94 protein structures and 1.43 Å, 0.68 Å and 1.70 Å for the clean set of 49 structures (Table S6). Those should be compared to the theoretical limit of accuracy, which is dictated by the best-rotameric structure (which has an average RMSD of 0.4 Å), and by the deviation in the same structure solved multiple times (which is 0.5, 1.0 and 0.8 Å for buried, exposed and all residues, respectively) [42]. Thus, for buried residues the lower bound on the side chain prediction accuracy expected to be ~0.64 Å RMSD (variance of the sum of the two independent factors mentioned above), which does not leave much room for improvement. This is illustrated in Figure 6, where all buried residues of *barstar *were perfectly modelled. The surface residues, which deviated in the model, also deviated in two *barstar *crystal structures or between the X-ray and NMR structures of *barnase*, showing the known tendency of surface residues to be flexible. Interestingly, when comparing the per amino acid type or per protein performance of Hunter to other methods, the different methods showed the same trends. Hydrophobic and aromatic amino acids are modelled best while polar amino acids perform worse. This is expected, as polar amino acids are more frequently located on the surface.

Hunter performs also as good or better than the top methods in comparative modelling when backbone coordinates are not accurate (Table 6). Particularly, the contact score (which evaluates the quality of predicted atom-atom contacts) was better for the Hunter-refined models than the submitted structures. While it is generally accepted that accuracy of the backbone coordinates affects accuracy of side chain placement, our results suggest that Hunter should be less sensitive to this effect and can absorb small backbone movement. This is due to the fact that side chain placement is driven by side chain interactions and thus less influenced by backbone conformation.

One of our future goals is to employ the 4-distance method for computational protein design, which is, in general, a two-step process. First, a detailed model of a protein or protein complex is built, and then, in the second step, its energetic characteristics are evaluated. At the moment Hunter is able to accurately model residue-residue contacts. The second step, however, would require dealing with additivity and independence of the terms as well as accounting for additional factors like interaction with water, entropic effects, and backbone flexibility and is the topic of future research.

## Conclusions

The Hunter knowledge-based potential uses a new 4-distance description of residue-residue interactions. We demonstrate that the statistical preferences on the 4-distance geometry can be extracted from high-resolution protein structures and applied to structure modelling. We show that Hunter is successful in identifying native protein structure among decoy structures and in accurate prediction of side chain conformations in protein structures. We describe and discuss all the necessary steps to construct the potential and possible alternatives for the choices being made. The supporting web site http://bioinfo.weizmann.ac.il/hunter/ is developed for the scientific community to make the results of our study easily accessible. The presented methodology described can be employed in other areas involving high-resolution modelling of biomolecules such as refinement of low-resolution structures, structure prediction, protein-protein docking and modelling mutations [43].

## Methods

### Knowledge-based potential

The knowledge-based potential was constructed as a linear combination of 4 terms:(1)

where *E*_*ScSc *_is a knowledge-based term based on detailed description of *ScSc *interaction geometry, *E*_*ScMc *_is a knowledge-based term based on detailed description of *ScMc *interaction geometry, *E*_*rot *_is the rotamer term, *E*_*lj *_is the Lennard-Jones term, and *w*_*ScSc*_, *w*_*ScMc*_, *w*_*rot*_, *w*_*lj *_are the weights for each term. The terms of the potential are detailed below.

### Side chain-side chain (*E*_*ScSc*_) and side chain-main chain (*E*_*ScMc*_) contact terms

The *E*_*ScSc *_contact term evaluates the geometry of *ScSc *interaction between pairs of residues. This geometry is defined in terms of 4 distances between two pairs of atoms; each pair is from a different residue (Figure 1). For every given pair of residues a specific set of 4 contact atoms is defined. As Gly does not have a side chain, only 190 (out of 210 possible) *ScSc *interactions were defined in the current study. To choose the set of 4 atoms, all possible combinations of 2 atoms per residue were defined. A precompiled set of 9394 high-resolution protein structures was obtained from PISCES server [44] (resolution < 2 Å, mutual sequence identity < 90%, R-value < 0.25) and the number of contacts between residues for each 4-atom combination was determined. Two residues were considered to be in contact if at least one of the 4 distances was less than 5 Å. For identifying real contacts, 5 Å is a relatively long cutoff, and may include cases where another piece of structure is in between. The final score (as described further in this section) is derived as log of the ratio *P*_*real*_/*P*_*rand*_. At about 5 Å little difference should be observed in inter-residue contacts derived from real and random structures, and those long-range contacts are mostly cancelled out [27]. In addition, as modelling is done using rotamer space, we extended the distance definition because of their discrete nature.

The set of 4 atoms, which gave the largest number of contacts for a given residue pair was chosen to define geometry of *ScSc *interaction (Table S1). Noteworthy, different pairs of atoms may be used to define the interaction with different residues. For example, NH1 and NH2 of Arg were chosen for the Arg-Lys pair, while CD2 and NH2 were chosen for the Arg-Asp pair (Table S1).

The collected contact data were used to build the real distribution - the probability of observing every 4-distance combination in high-resolution protein structures. To this end, a four-dimensional histogram with a constant bin size of 0.5 Å along each dimension from 0 to 10 Å was built. An additional step of smoothing was applied such that the bin value was recalculated as an average of values in adjacent bins and the bin itself. The number of measurements within a single bin was divided by the total number of measurements, thus the sum of all bin values equals to one.

Similarly, the random distribution was built based on contact data collected from randomized protein structures. These structures were obtained by modelling at each position a side chain rotamer picked at random from the backbone-dependent rotamer library (see details on generating the rotamer library below). All rotamers were treated equally without considering their probabilities and irrespective of rotamer clashes. Clashes do not interfere as the final score is derived as the ratio of *P*_*real*_/*P*_*rand*_, and thus a large *P*_*rand *_value would only increase the unfavourable score for clashing distances. The identities of amino acids during modelling were preserved. The set of randomized structures was build based on the list of 9394 proteins described above.

The equation to calculate the *E*_*ScSc *_term is given as follows:(2)

where *M *is a number of residues, *P*_*real *_and *P*_*rand *_are the product of two probabilities:(3)

*P*({*dist*}|*AA*) is the probability of observing the 4-distance combination for a given residue pair and *P*(*AA*) is the probability to observe a *ScSc *contact for a given residue pair in protein structures. High-resolution protein structures and random structures were used to derive *P*_*real *_and *P*_*rand*_, respectively. The summation is done over all unique contacting residue pairs. In a similar way, the *E*_*ScMc *_term was constructed to account for the *ScMc *interaction geometry (see Additional file 1: Supplementary Methods for complete details).

### Rotamer term (*E*_*rot*_)

The rotamer term was incorporated to the potential to penalize for side chain conformations with low probability and is defined as follows:(4)

where  is the rotamer probability as taken from the backbone-dependent rotamer library [45],  is a number of rotamers for a modelled residue, and *M *is a number of residues.

### Lennard-Jones term (*E*_*lj*_)

The packing of atoms in the protein structure is modelled with the Lennard-Jones term as follows:(5)

where *ε*_*ij *_is the depth of the potential well,  is the distance at the minimum of the potential, and *d*_*ij *_is the distance between two atoms. The favourable energies were accumulated in the *E*_*lja *_term and the repulsive ones in the *E*_*ljr *_term. To avoid excessive repulsion due to close placement of atoms during side chain optimization, the repulsive term *E*_*ljr *_was linearized at a cutoff distance *d*_*ij *_< 0.89 [46]. All protein heavy atoms in the Lennard-Jones term calculations were grouped into 20 classes (Table S9). The well depths for a pair of atoms were calculated as , where individual *ε*_*i *_values for each atom class were defined as in the CHARMM19 parameter set [47]. The radii in calculating  = *r*_*i *_+ *r*_*j *_distances were fitted to reproduce interatomic distances observed in protein structures. To perform the fit, PROBE software was used to determine atoms in direct contact [35]. PROBE was run with an increased probe radius of 1 Å (using "-Radius1.0" flag) assuming implicit hydrogens ("-Implicit"). Mainchain-mainchain interactions were included in the calculations ("-MC"). The unformatted PROBE output (generated with "-Unformatted" flag) was processed to extract all pairs of atoms in direct contact in the set of 9394 high-resolution protein structures. Then the interatomic distances were determined from the corresponding PDB structures. Distance distributions were built for all 210 types of pairwise interatomic distances. The peak location in each distribution was used to identify the optimal interactomic distances. No special consideration was given to different geometries of interacting atoms (e.g. edge and face distances for aromatic C). For polar atoms only the distances to non-polar atoms were used to define the radii, to avoid the shorter distances found in hydrogen bonds. To model correctly polar-polar interactions, the optimal distance between them in the LJ term was set to reproduce those found in the PDB. Some other types were discarded as not having a sufficient number of observations (< 10000). The least-squares fitting of 20 atom radii was performed for the remaining 91 pairwise distances with results presented in Table S9.

### Rotamer library

A discrete set of side chain conformations from the backbone-dependent rotamer library was used to rebuild side chains [45]. The original rotamer library was extended by adding additional rotamers, whose chi-angles deviate by one sigma from the tabulated values. Thus, a rotamer in the original library with *n *dihedral angles is replaced in the extended library by *3*^*n*^. The rotamer probabilities were recalculated assuming a normal distribution around the tabulated chi-angles with the variances given in the library. In addition, a reduced library was created by ranking rotamers by probability and discarding low-probability rotamers (with a cumulative probability of 0.03). This decreased the library size by half while maintaining reasonable sampling accuracy. Both libraries were tested for side chain modelling and are referred in the text as "full" and "reduced" libraries.

### Side chain placement

Monte Carlo Simulated Annealing (MCSA) method is used to optimize side chain conformations [34]. Initially, random rotamers were assigned to all side chains, with the temperature being gradually raised till 95% of the moves were accepted. Each MC move comprises of choosing the position and the rotamer to be placed at that position. Every position and every rotamer are sampled uniformly at random. The rotamers are chosen from the backbone-dependent rotamer library taking into account the φ/ϕ backbone angles of the modelled position. If a new state gets a lower score (*E*_*new*_) than the previous one (*E*_*0*_) the move is accepted. Otherwise, the move is accepted with Boltzmann probability *p *= exp(-Δ*E*/*T*), where Δ*E *= *E*_*new*_- *E*_0_. During MC cycle the temperature was gradually decreased to zero in 100 steps with 10000 rotamer substitutions at each step. The structure with the lowest score was tracked during the optimization. At the final stage, a quenching was performed: a position is picked at random and every rotamer was tested at that position. If a new rotamer lowered the score, it was kept; otherwise, it was rejected. The procedure was repeated many times till the moment that any single rotamer at any position does not improve the score further.

### Fitting weights

The relative contribution of different terms in the KBP was parameterized on a set of 30 proteins (PDB IDs: 1iib, 1bqk, 1d7p, 1bkr, 1nwp, 1svy, 1vhh, 1nbc, 1nkr, 1onc, 1xnb, 3std, 1kuh, 1m6p, 1c52, 1evh, 1vsr, 1mug, 2tnf, 1qft, 1flp, 1ido, 1mgt, 1cv8, 5cyt, 1npk, 1atz, 1dhn, 2cua, 1tx4) from the Protein Data Bank [48]. These proteins share less than 30% sequence identity with each other and with any protein in the test set (see Results). The weights were calibrated in order to minimize the root mean square deviation (RMSD) of side chain atoms between the model and the native structure (note that similar weights were obtained when fitting using a smaller (10 proteins) or larger (100 proteins) training set). In the first step, the relative contribution of *E*_*rot *_and *E*_*lj *_terms were determined by varying the parameter λ from 0 to 1 with 0.05 steps in the equation *E *= λ*E*_*rot*_+ (1-λ)*E*_*lj*_. Then, *E*_*ScSc *_term was weighted similarly relative to *E*_*rot *_and *E*_*lj *_considering them as a single term. At the last step, *E*_*ScMc *_was weighted relative to other terms. The final set of weights was 0.13, 0.13, 0.33, 0.41 for *w*_*ScSc*_, *w*_*ScMc*_, *w*_*rot*_, *w*_*lj*_, respectively. Additional optimization was applied such that each weight was modified by small increment/decrement at a time to identify a better nearby solution. However, the improvement in side chain modelling accuracy was negligible.

### Contact score

The contact score [49] was used to evaluate the accuracy of *ScSc *and *ScMc *contact prediction. First, all *ScSc *and *ScMc *atom-atom contacts were determined in the native protein structure. Two atoms were considered to be in contact if the distance between their centers was less than sum of van der Waals radii plus 1 Å. The van der Waals radii used in the calculation were 1.548 Å for C, 1.348 Å for O, 1.400 Å for N, and 1.808 Å for S. Then contacts were calculated in the modelled structure. Those that had equivalent contacts in the native structure were given a score. If the contact in the modelled structure was between 0.125 Å further apart and 0.0675 Å closer than the equivalent contact in the native structure, a score of 4 was assigned. If the contact did not fall within this interval but was within the next interval 0.25 Å/0.125 Å, a score of 3 was assigned and, further on, a score of 2 for 0.5 Å/0.25 Å interval, a score of 1 for 1 Å/0.5 Å interval. Total score was divided by the number of native contacts and multiplied by 25 to scale it from 0 to 100.

## Authors' contributions

VP and MC carried out the study and drafted the manuscript. YI worked on computational optimization of the method. GS conceived the study, and participated in its design and coordination and helped to draft the manuscript. All authors read and approved the final manuscript.

## Supplementary Material

Additional file 1 Supplementary Methods and Tables Click here for file
