# Supplementary material for: Protein structure modelling and evaluation based on a 4-distance description of side-chain interactions
Source: BMC Bioinformatics. 2010 Jul 12;11:374. doi: 10.1186/1471-2105-11-374 (PMC2912888; doi:10.1186/1471-2105-11-374)
Supplement: Additional file 1 — Supplementary Methods and Tables [file 1471-2105-11-374-S1.PDF]

Protein structure modelling and evaluation based on a 4-distance description of side-chain interactions

Vladimir Potapov, Mati Cohen, Yuval Inbar and Gideon Schreiber

Supplementary methods:

- Side chain-main chain contact term (E_{ScMc})

Supplementary tables:

- **Table S1.** Identities of the side chain atoms in "Atom Set 1" defining 190 ScSc inter-residue interactions
- **Table S2.** Identities of the side chain atoms in "Atom Set 1" defining 18 ScMc inter-residue interactions
- **Table S3.** Identities of the side chain atoms in "Atom Set 2" defining 190 ScSc inter-residue interactions
- **Table S4.** Assessment of four scoring functions for discrimination of the native structure among Decoys 'R' Us multiple decoys sets
- **Table S5.** Assessment of four scoring functions for discrimination of the native structure among CASP 7/8 multiple decoys sets
- **Table S6.** Side chain prediction accuracy with Hunter for a set of 94 high-resolution protein structures
- **Table S7.** Normalization factors for 20 different amino acids
- **Table S8.** Interface remodeling for 20 protein-protein hetero-complexes
- **Table S9.** The parameter set used in the Lennard-Jones term calculation

Supplementary Methods

Side chain-main chain contact term (E_{ScMc})

The E_{ScMc} term was constructed in a similar way as the E_{ScSc} term except changes were made in choosing the four atoms. Two side chain atoms in the first residue and two backbone atoms in the second one define residue-residue interaction in the E_{ScMc} term. Firstly, Cα and backbone oxygen were chosen as two backbone atoms and were kept fixed for all residue pairs. These two atoms were found to interact more often among other backbone atoms in our analysis of side chain to main chain contacts. Then, side chain atoms were identified by examining all possible side chain atom pairs. Those that gave the largest number of contacts with the chosen backbone atoms in 9394 high-resolution protein structures were used in the calculations (Table S2). Main chain atoms in *ScMc* interactions were treated irrespective of the residue type. Therefore, 18 *ScMc* interactions were defined. As Ala does not have a flexible side chain and Gly does not have a side chain at all, those residues were not used to define side chain interactions with main chain atoms.

To build the random distributions, the same randomized structures as for the *ScSc* term were used since no random control could be really done for the main chain. Probabilities from these distributions were used to calculate the E_{ScMc} term as follow:

$$E_{ScMc} = \sum_{i}^{M} \sum_{j, j \neq i, j \neq i+1}^{M} -\ln \frac{P_{real}(\{dist\} \mid AX) P_{real}(AX)}{P_{rand}(\{dist\} \mid AX) P_{rand}(AX)}$$

where AX denotes an interaction of side chain atoms of the first residue with main chain atoms of the second residue; $P_{real}(\{dist\} \mid AX)$ and $P_{rand}(\{dist\} \mid AX)$ are the probabilities of observing the *ScMc* 4-distance combination $\{dist\}$ in high-resolution and random structures, respectively; $P_{real}(AX)$ and $P_{rand}(AX)$ are the probabilities to observe a *ScMc* contact for residue A in real and random structures, respectively; and M is a number of residues. The

summation is done over all contacting residue pairs excluding within-residue interactions and *ScMc* interaction between neighboring residues.

Supplementary Tables

Table S1 - Identities of the side chain atoms in “Atom Set 1” defining 190 ScSc inter-residue interactions

Each cell of the table lists two pairs of atoms that define interaction for a particular residue pair: the first atom pair corresponds to a residue in the rightmost column, the second atom pair corresponds to a residue in the top row. The number in each cell gives the number of *ScSc* contacts extracted for a given residue pair from a set of high-resolution protein structures (see Methods for details).

ALA	ARG	ASN	ASP	CYS	GLN	GLU	HIS	ILE	LEU	LYS	MET	PHE	PRO	SER	THR	TRP	TYR	VAL	
CA,CB<br>CA,CB<br>28828	CA,CB<br>CB,CD<br>16383	CA,CB<br>CB,ND2<br>15236	CA,CB<br>CB,OD1<br>18605	CA,CB<br>CB,SG<br>8032	CA,CB<br>OE1,NE2<br>11327	CA,CB<br>CB,OE2<br>18645	CA,CB<br>CB,CE1<br>10445	CA,CB<br>CG2,CD1<br>46439	CA,CB<br>CD1,CD2<br>74802	CA,CB<br>CB,CE<br>14794	CA,CB<br>CG,CE<br>12753	CA,CB<br>CE1,CE2<br>28729	CA,CB<br>CB,CD<br>18679	CA,CB<br>CB,OG<br>22854	CA,CB<br>CG2,OG1<br>26484	CA,CB<br>CG,CE3<br>6916	CA,CB<br>CD1,OH<br>17879	CA,CB<br>CG2,CG1<br>53499	ALA
	CG,NH2<br>CD,NH2<br>13711	CG,NH2<br>OD1,ND2<br>11813	CD,NH2<br>OD1,OD2<br>32825	CG,NH2<br>CB,SG<br>3163	CG,NH2<br>OE1,NE2<br>10914	CD,NH2<br>OE1,OE2<br>37015	CG,NH2<br>ND1,NE2<br>6662	CG,NH2<br>CG2,CD1<br>15948	CG,NH2<br>CD1,CD2<br>28532	NH1,NH2<br>CB,NZ<br>8002	CG,NH2<br>SD,CE<br>5249	CG,NH2<br>CE1,CE2<br>12655	NH1,NH2<br>CB,CD<br>10891	CG,NH2<br>CB,OG<br>14065	CG,NH2<br>CG2,OG1<br>14509	CG,NH2<br>CD1,CH2<br>6513	CG,NH2<br>CE1,OH<br>14352	CG,NH2<br>CG2,CG1<br>18247	ARG
		CB,OD1<br>OD1,ND2<br>13829	OD1,ND2<br>OD1,OD2<br>16094	CB,OD1<br>CB,SG<br>2873	OD1,ND2<br>OE1,NE2<br>8893	OD1,ND2<br>OE1,OE2<br>12534	OD1,ND2<br>ND1,NE2<br>5388	OD1,ND2<br>CG2,CD1<br>11124	CB,ND2<br>CD1,CD2<br>15813	OD1,ND2<br>CG,NZ<br>11936	CB,ND2<br>CB,CE<br>3916	OD1,ND2<br>CG,CZ<br>8522	OD1,ND2<br>CB,CD<br>8613	OD1,ND2<br>CB,OG<br>13669	OD1,ND2<br>CG2,OG1<br>14057	CB,ND2<br>NE1,CZ3<br>4307	OD1,ND2<br>CE1,OH<br>8885	CB,ND2<br>CG2,CG1<br>12391	ASN
			CB,OD2<br>OD1,OD2<br>14135	CB,OD1<br>CB,SG<br>3117	OD1,OD2<br>OE1,NE2<br>10323	OD1,OD2<br>OE1,OE2<br>10960	OD1,OD2<br>ND1,NE2<br>10629	CB,OD2<br>CG2,CD1<br>11630	CB,OD2<br>CD1,CD2<br>17933	OD1,OD2<br>CG,NZ<br>29305	CB,OD2<br>CG,CE<br>4087	CB,OD1<br>CE1,CE2<br>8452	CB,OD1<br>CB,CD<br>9789	OD1,OD2<br>CB,OG<br>22088	OD1,OD2<br>CG2,OG1<br>19469	CB,OD1<br>NE1,CH2<br>4768	OD1,OD2<br>CE1,OH<br>12933	CB,OD2<br>CG2,CG1<br>14190	ASP
				CB,SG<br>CB,SG<br>7400	CB,SG<br>CB,NE2<br>2400	CB,SG<br>CG,OE1<br>2575	CB,SG<br>ND1,NE2<br>2438	CB,SG<br>CG2,CD1<br>8570	CB,SG<br>CD1,CD2<br>13580	CB,SG<br>CB,CE<br>2461	CB,SG<br>CG,CE<br>2902	CB,SG<br>CE1,CE2<br>6452	CB,SG<br>CB,CD<br>4315	CB,SG<br>CB,OG<br>4515	CB,SG<br>CG2,OG1<br>4558	CB,SG<br>CB,CH2<br>2694	CB,SG<br>CE1,OH<br>3174	CB,SG<br>CG2,CG1<br>9776	CYS
					CG,OE1<br>OE1,NE2<br>7240	OE1,NE2<br>OE1,OE2<br>10013	OE1,NE2<br>ND1,NE2<br>4456	CB,NE2<br>CG2,CD1<br>11188	CB,NE2<br>CD1,CD2<br>19151	OE1,NE2<br>CG,NZ<br>9244	CB,NE2;<br>CG,CE<br>3758	CB,NE2<br>CE1,CE2<br>8478	OE1,NE2<br>CB,CD<br>7787	OE1,NE2<br>CB,OG<br>9979	OE1,NE2<br>CG2,OG1<br>10958	CB,NE2<br>NE1,CH2<br>4382	OE1,NE2<br>CE1,OH<br>7787	CB,NE2<br>CG2,CG1<br>12149	GLN
						CG,OE2<br>OE1,OE2<br>11259	OE1,OE2<br>ND1,NE2<br>10120	CB,OE2<br>CG2,CD1<br>15629	CG,OE1<br>CD1,CD2<br>25176	OE1,OE2<br>CD,NZ<br>32578	CG,OE1<br>CG,CE<br>4764	CB,OE2<br>CE1,CE2<br>11153	OE1,OE2<br>CB,CG<br>10493	OE1,OE2<br>CB,OG<br>16944	OE1,OE2<br>CG2,OG1<br>17036	CG,OE1<br>NE1,CH2<br>5650	OE1,OE2<br>CE1,OH<br>13679	CB,OE2<br>CG2,CG1<br>16990	GLU
							ND1,NE2<br>ND1,NE2<br>3473	ND1,NE2<br>CG2,CD1<br>7430	ND1,NE2<br>CD1,CD2<br>13065	ND1,NE2<br>CG,NZ<br>4123	ND1,NE2<br>SD,CE<br>2967	ND1,NE2<br>CE1,CE2<br>6588	ND1,NE2<br>CB,CD<br>6371	ND1,NE2<br>CB,OG<br>7826	ND1,NE2<br>CG2,OG1<br>7480	ND1,NE2<br>CD1,CH2<br>3301	ND1,NE2<br>CE1,OH<br>7342	ND1,NE2<br>CG2,CG1<br>8788	HIS
								CB,CD1<br>CG2,CD1<br>56288	CG2,CD1<br>CD1,CD2<br>104267	CG2,CD1<br>CB,CE<br>13759	CG2,CD1<br>SD,CE<br>15528	CG2,CD1<br>CE1,CE2<br>38895	CG2,CD1<br>CB,CD<br>16563	CG2,CD1<br>CB,OG<br>17193	CG2,CD1<br>CG2,OG1<br>24164	CG2,CD1<br>CB,CH2<br>11831	CG2,CD1<br>CE1,OH<br>18168	CG2,CD1<br>CG2,CG1<br>62218	ILE
									CG,CD1<br>CD1,CD2<br>149892	CD1,CD2<br>CB,CE<br>21197	CD1,CD2<br>SD,CE<br>25728	CD1,CD2<br>CE1,CE2<br>67488	CD1,CD2<br>CB,CD<br>27200	CD1,CD2<br>CB,OG<br>25193	CD1,CD2<br>CG2,OG1<br>36221	CD1,CD2<br>CB,CH2<br>20814	CD1,CD2<br>CE1,OH<br>30748	CD1,CD2<br>CG2,CG1<br>106735	LEU
										CB,NZ<br>CG,NZ<br>5914	CB,CE<br>SD,CE<br>4175	CB,CE<br>CE1,CE2<br>10293	CB,NZ<br>CB,CD<br>6546	CB,NZ<br>CB,OG<br>12679	CG,NZ<br>CG2,OG1<br>11890	CB,CE<br>CD1,CH2<br>4544	CG,NZ<br>CE1,OH<br>12277	CB,CE<br>CG2,CG1<br>14462	LYS
											CG,CE<br>SD,CE<br>6198	SD,CE<br>CE1,CE2<br>12219	SD,CE<br>CB,CD<br>5647	CG,CE<br>CB,OG<br>5324	CG,CE<br>CG2,OG1<br>7155	CG,CE<br>CB,CH2<br>4330	CG,CE<br>CE1,OH<br>6655	CG,CE<br>CG2,CG1<br>16073	MET
												CE1,CE2<br>CG,CZ<br>31175	CE1,CE2<br>CB,CD<br>15125	CE1,CE2<br>CB,OG<br>12800	CE1,CE2<br>CG2,OG1<br>15218	CE1,CE2<br>CB,CH2<br>10429	CE1,CE2<br>CE1,OH<br>15052	CE1,CE2<br>CG2,CG1<br>38808	PHE
													CB,CG<br>CB,CD<br>9459	CB,CD<br>CB,OG<br>11851	CB,CD<br>CG2,OG1<br>13074	CB,CD<br>CD1,CH2<br>8270	CB,CD<br>CE1,OH<br>15382	CB,CD<br>CG2,CG1<br>20311	PRO
														CB,OG<br>CB,OG<br>9804	CB,OG<br>CG2,OG1<br>18332	CB,OG<br>CD1,CH2<br>5786	CB,OG<br>CE1,OH<br>10451	CB,OG<br>CG2,CG1<br>20169	SER
															CB,CG2<br>CG2,OG1<br>17162	CG2,OG1<br>CD1,CH2<br>5936	CG2,OG1<br>CE1,OH<br>11118	CG2,OG1<br>CG2,CG1<br>27061	THR
																CB,CH2<br>CD1,CH2<br>4411	CD1,CH2<br>CE1,OH<br>5695	CB,CH2<br>CG2,CG1<br>12617	TRP
																	CE1,OH<br>CE1,OH<br>4759	CE1,OH<br>CG2,CG1<br>18718	TYR
																		CB,CG2<br>CG2,CG1<br>55481	VAL

Table S2 - Identities of the side chain atoms in “Atom Set 1” defining 18 ScMc inter-residue interactions

The ScMc interaction between residues was defined by two pairs of atoms: one atom pair is chosen on the side chain of the first residue, the other atom pair is chosen on the backbone of the second residue. The side chain atom pairs are shown in the table. The backbone atom pairs were kept fixed for all residues (alpha carbon and backbone oxygen).

	Constrained atoms	Number of contacts
**ARG**	CG, NH2	264471
**ASN**	OD1, ND2	199717
**ASP**	OD1, OD2	231103
**CYS**	CB, SG	90448
**GLN**	OE1, NE2	151895
**GLU**	CB, OE2	236017
**HIS**	ND1, NE2	102061
**ILE**	CG2, CD1	326939
**LEU**	CD1, CD2	386244
**LYS**	CB, NZ	240001
**MET**	CB, CE	110253
**PHE**	CE1, CE2	216867
**PRO**	CB, CD	243007
**SER**	CB, OG	279342
**THR**	CG2, OG1	281782
**TRP**	NE1, CZ3	95316
**TYR**	CE1, CE2	177598
**VAL**	CG2, CG1	381847

Table S3 - Identities of the side chain atoms in “Atom Set 2” defining 190 ScSc inter-residue interactions

The pair of side chain atoms was manually picked for each of 20 amino acids. The interaction of two residues was defined by the four distances between two atom pairs. The atom pair for a given residue type is kept fixed. The pair of atoms for each residue type is given in the column “Constrained atoms”. Numbers for each residue pair show amount of *ScSc* contacts identified in high-resolution protein structures (see Methods for details).

	Constrained atoms	ALA	ARG	ASN	ASP	CYS	GLN	GLU	HIS	ILE	LEU	LYS	MET	PHE	PRO	SER	THR	TRP	TYR	VAL
**ALA**	CA, CB	28828																		
**ARG**	NH1, NH2	16383	5676																	
**ASN**	OD1, ND2	15236	9913	6852																
**ASP**	OD1, OD2	18605	31262	16094	7533															
**CYS**	SG, CB	8032	2271	2726	2761	7400														
**GLN**	OE1, OE2	11327	9258	8893	10323	2156	3607													
**GLU**	OE1, OE2	18645	35342	12534	10960	2284	10013	5685												
**HIS**	ND1, NE2	10445	5905	5388	10629	2438	4456	10120	3473											
**ILE**	CG2, CD1	46439	11568	11124	10400	8570	10121	12335	7430	33285										
**LEU**	CD1, CD2	74802	21895	15829	16513	13580	17878	21679	13065	104267	96776									
**LYS**	CD, NZ	14794	6826	10882	27530	1804	8424	32578	3883	10795	17426	2539								
**MET**	SD, CE	12753	3703	3397	2979	2739	3050	3681	2967	15528	25728	3329	3080							
**PHE**	CE1, CE2	28729	9478	8037	7166	6452	7690	9106	6588	38895	67488	8361	12219	16140						
**PRO**	CB, CD	18679	10891	8613	9456	4315	7787	10526	6371	16563	27200	5517	5647	15125	4978					
**SER**	CB, OG	22854	11722	13669	22088	4515	9979	16944	7826	17193	25193	10002	4896	12800	11851	9804				
**THR**	OG1, CG2	26484	11706	14057	19469	4558	10958	17036	7480	24164	36221	10274	6784	15218	13074	18332	9722			
**TRP**	CE3, NE1	6916	4607	3862	4097	1973	3775	4664	3051	9614	16936	3527	3465	8329	7399	5185	5359	1566		
**TYR**	CE1, OH	17879	11522	8885	12933	3174	7787	13679	7342	18168	30748	11760	6315	15052	15382	10451	11118	4942	4759	
**VAL**	CG1, CG2	53499	13552	12128	12327	9776	11170	13935	8788	62218	106735	11552	16012	38808	20311	20169	27061	10568	18718	35567

Table S4 - Assessment of four scoring functions for discrimination of the native structure among Decoys 'R' Us multiple decoys sets

The rank of the native structure is given in the columns. Hunter scores for each decoy structure were calculated using the *ScSc* term only. The results for OPUS-PSP were taken from Lu et al. [1]. The results for DOPE and DFIRE were taken from Shen et al. [2]. Resolution is given for the native structure.

	Resolution Å	Hunter	OPUS-PSP	DOPE	DFIRE
4state_reduced					
1sn3	1.20	1	1	1	1
4rxn	1.20	1	1	1	1
4pti	1.50	1	1	1	1
1ctf	1.70	1	1	1	1
1r69	2.00	1	1	1	1
3icb	2.30	15	1	1	4
2cro	2.35	1	1	1	1
fisa					
4icb	1.60	1	1	1	1
2cro	2.35	1	1	1	1
1fc2	2.80	33	312	375	254
1hdd-C	2.80	1	1	1	1
fisa_casp3					
smd3	?	1	1	-	-
1bg8-A	2.20	1	1	1	1
1bl0	2.30	1	1	1	1
1eh2	NMR	1	1	-	-
1jwe	NMR	1	1	1	1
lattice_ssfit					
4icb	1.60	1	1	1	1
1ctf	1.70	1	1	1	1
1fca	1.80	1	1	1	1
1pgb	1.92	1	1	1	1
1beo	2.20	1	1	1	1
1dkt-A	2.90	1	1	1	1
1nkl	NMR	1	1	1	1
1trl-A	NMR	1	1	1	1
lmds					
1igd	1.10	1	1	1	1
2ovo	1.50	1	1	1	1
4pti	1.50	1	1	1	1
1ctf	1.70	1	1	1	1
1b0n-B	1.90	1	1	34	430
1shf-A	1.90	1	1	1	1
2cro	2.35	1	1	1	1
1fc2	2.80	49	409	476	501
1bba	NMR	501	501	501	501
1dtk	NMR	3	1	1	1
**Correct predictions**		**29**	**31**	**28**	**27**

Table S5 - Assessment of four scoring functions for discrimination of the native structure among CASP 7/8 multiple decoys sets

Numbers of in the columns for Hunter, OPUS-PSP, DOPE and DFIRE methods give the rank of the native structure. Hunter scores for each decoy structure were calculated using the *ScSc* term only. Three methods (OPUS-PSP, DOPE, DFIRE) were chosen for comparison among other methods as they have been successfully applied in the past for discriminating native structures in decoys sets, and the respective programs are readily accessible for performing calculations. In the end of table, the overall number of correct predictions is given, as well as the number of correct prediction considering high-resolution (resolution < 2 Å) decoys only.

	Resolution Å	Hunter	OPUS-PSP	Rosetta	DOPE	DFIRE
CASP7						
T0288	1.1	2	1	85	8	4
T0359	1.4	1	1	1	15	1
T0340	1.5	1	1	5	8	7
T0324_D1	1.5	1	1	1	1	2
T0324_D2	1.5	1	1	2	2	3
T0305	1.6	1	1	1	1	1
T0332	1.6	1	1	1	1	1
T0366	1.7	1	1	2	3	9
T0291	1.8	1	1	3	1	1
T0290	1.8	2	1	10	1	1
T0313	1.9	1	1	1	1	1
T0311	1.9	1	1	6	1	3
T0295_D1	1.9	1	1	2	1	1
T0295_D2	1.9	1	1	1	3	1
T0303_D1	1.9	1	1	1	1	1
T0308	2.0	1	1	6	1	1
T0317	2.0	1	1	1	1	1
T0346	2.0	6	1	2	8	12
T0339_D2	2.1	1	1	1	1	1
T0345	2.1	1	1	1	1	1
T0367	2.2	1	1	6	1	5
T0292_D1	2.2	4	1	11	13	15
T0292_D2	2.2	1	1	2	1	1
T0315	2.2	1	1	3	1	1
T0334	2.5	1	1	1	1	1
T0326	2.5	1	1	1	1	1
T0328	2.8	1	1	12	1	1
CASP8						
T0488-D1	1.3	1	1	8	1	2
T0508-D1	1.5	1	1	6	1	1
T0459-D1	1.7	1	1	10	13	21
T0423-D1	1.7	1	1	51	1	1
T0454-D1	1.8	22	69	42	14	61
T0504-D3	1.8	1	1	2	1	2
T0445-D1	1.8	1	1	1	1	1

T0392-D1	1.8	1	1	11	7	10
T0447-D1	1.9	1	1	1	1	1
T0505-D1	1.9	1	1	2	1	1
T0506-D1	1.9	1	1	8	1	1
T0432-D1	1.9	1	1	3	1	1
T0388-D1	2.0	1	1	1	1	1
T0402-D1	2.0	1	1	3	1	1
T0418-D1	2.0	1	1	13	1	20
T0418-D2	2.0	1	1	5	2	1
T0422-D2	2.0	1	1	1	1	10
T0491-D1	2.0	12	1	12	13	12
T0428-D1	2.0	3	7	10	8	10
T0426-D1	2.1	3	1	9	6	5
T0396-D1	2.1	1	1	12	1	1
T0398-D1	2.1	1	1	1	3	14
T0398-D2	2.1	1	1	2	2	3
T0453-D1	2.1	3	1	14	5	24
T0435-D1	2.2	1	1	11	1	1
T0400-D1	2.2	1	1	6	1	5
T0452-D1	2.2	1	1	2	1	1
T0452-D2	2.2	1	1	1	1	1
T0486-D1	2.3	1	1	5	1	11
T0404-D1	2.4	1	1	9	1	1
T0479-D1	2.4	4	3	61	9	84
T0456-D2	2.5	2	1	6	1	2
T0390-D1	2.7	1	1	8	2	2
T0455-D1	2.7	1	1	69	7	36
T0416-D1	2.7	1	2	29	3	14
T0450-D1	2.7	3	1	9	2	15
T0458-D1	2.7	7	38	92	6	39
T0438-D1	2.8	5	3	59	4	23
T0438-D2	2.8	11	62	184	133	181
T0442-D1	2.8	11	31	76	78	126
T0442-D2	2.8	66	159	9	161	184
T0444-D1	2.8	7	11	70	17	97
T0461-D1	2.8	1	1	6	1	5
T0441-D2	2.9	1	1	43	1	19
T0470-D1	2.9	36	64	296	188	269
T0470-D2	2.9	16	9	128	165	225
Correct predictions (all)		**53**	**61**	**18**	**42**	**33**
Correct predictions (R < 2 Å)		**24**	**26**	**9**	**18**	**16**

Table S6 - Side chain prediction accuracy with Hunter for a set of 94 high-resolution protein structures

The average side chain RMSD over a set of 94 structures is 1.47 Å for all residues (0.73 Å for buried residues). A star next to PDB ID indicates structures that contain large ligands or are a part of larger macromolecular assembly. The average RMSD over a set 49 "clean" structures is 1.43 Å (0.68 Å for buried residues) and 1.51 Å (0.79 Å for buried residues) for other 45 structures. RMSD was calculated for side chain atoms (beyond Cβ). The complete rotamer library was used (see Methods for details).

PDB ID	Resolution Å	RMSD, Å			Chain Length
		All	Buried	Exposed	
1aac	1.31	1.03	0.60	1.24	105
1amm	1.20	1.46	0.55	1.76	174
1arb	1.20	1.03	0.98	1.07	263
1ben	1.40	1.70	0.72	1.85	51
1bpi	1.09	1.36	0.54	1.56	58
1cem	1.65	1.09	0.74	1.37	363
1cnv	1.65	1.62	0.65	2.10	283
1cus	1.25	1.28	0.69	1.59	197
1ezm	1.50	1.35	0.57	1.73	298
1fus	1.30	1.48	0.51	1.90	105
1hfc	1.50	1.39	0.63	1.71	157
1ifc	1.19	1.64	0.80	1.99	131
1igd	1.10	1.20	0.43	1.32	61
1lit	1.55	1.33	0.45	1.65	131
1mla	1.50	1.53	0.75	1.92	305
1mrj	1.60	1.41	0.73	1.77	247
1osa	1.68	1.51	0.49	1.69	148
1php	1.65	1.54	0.81	1.83	394
1plc	1.33	1.17	0.55	1.45	99
1poa	1.50	1.71	0.62	1.97	118
1ptf	1.60	1.62	0.47	1.93	87
1ptx	1.30	1.82	0.37	1.96	64
1rcf	1.40	1.28	0.79	1.55	169
1rie	1.50	1.26	0.47	1.55	127
1rro	1.30	1.55	0.74	1.83	108
1smd	1.60	1.40	1.16	1.63	495
1snc	1.65	1.94	0.63	2.24	135
1tca	1.55	1.04	0.58	1.34	317
1xyz	1.40	1.08	0.67	1.36	320
2ayh	1.60	1.13	0.81	1.31	214
2cba	1.54	1.43	0.61	1.79	258
2cpl	1.63	1.46	0.66	1.79	164
2ctc	1.40	1.16	0.78	1.43	307
2end	1.45	1.53	0.62	1.81	137
2ihl	1.40	1.54	0.54	1.84	129

2mcm	1.50	1.20	0.46	1.39	112
2mhr	1.30	1.60	1.34	1.73	118
2phy	1.40	1.59	0.79	1.96	125
2rn2	1.48	1.83	0.68	2.14	155
2trx	1.68	1.40	0.55	1.65	108
2wrp	1.65	1.94	0.76	2.37	210
3chy	1.66	1.47	0.65	1.76	128
3ebx	1.40	1.42	0.76	1.54	62
3lzm	1.70	1.53	0.96	1.72	164
3pte	1.60	1.08	0.62	1.37	347
4fgf	1.60	1.69	0.84	1.93	124
4ptp	1.34	1.32	0.63	1.66	223
7rsa	1.26	1.52	0.67	1.77	124
8abp	1.49	1.30	0.82	1.56	305
1ads*	1.65	1.59	1.03	1.92	315
1aky*	1.63	1.32	0.72	1.52	218
1aru*	1.60	1.30	0.86	1.60	336
1bkf*	1.60	1.63	1.21	1.75	107
1cka*	1.50	1.44	0.48	1.71	66
1cpc*	1.66	1.42	0.71	1.57	172
1cse*	1.20	1.06	0.78	1.18	337
1ctj*	1.10	1.41	0.52	1.62	89
1dad*	1.60	1.48	0.89	1.76	224
1dif*	1.70	1.89	0.68	2.30	198
1fnc*	2.00	1.42	0.73	1.71	296
1fxd*	1.70	1.67	0.79	1.78	58
1iro*	1.10	1.18	0.46	1.35	53
1isu*	1.50	1.22	0.41	1.35	62
1jbc*	1.15	1.27	0.78	1.52	237
1kap*	1.64	1.49	1.08	1.82	474
1knb*	1.70	1.34	0.62	1.65	186
1lam*	1.60	1.42	0.85	1.72	484
1lkk*	1.00	1.60	0.48	1.87	109
1luc*	1.50	1.60	0.98	1.92	320
1nfp*	1.60	1.30	0.83	1.51	228
1nif*	1.70	1.53	0.79	1.88	333
1phb*	1.60	1.48	0.79	1.88	405
1ra9*	1.55	1.36	0.65	1.60	159
1rge*	1.15	1.36	0.81	1.58	96
1rpo*	1.40	1.61	0.95	1.80	122
1sgp*	1.40	1.79	1.10	1.98	51
1sri*	1.65	1.86	0.97	2.18	118
1tta*	1.70	1.46	0.31	1.68	127
1whi*	1.50	2.17	1.11	2.42	122
1xic*	1.60	1.95	1.23	2.27	385
1xso*	1.49	1.33	0.80	1.56	150
256b*	1.40	1.85	0.40	2.07	106
2bop*	1.70	1.96	0.71	2.14	85
2ccy*	1.67	1.57	0.55	1.78	127
2er7*	1.60	1.06	0.64	1.38	336
2hft*	1.69	1.44	0.69	1.70	205
2msb*	1.70	1.25	0.71	1.43	111
2olb*	1.40	1.30	0.86	1.67	520

2rhe*	1.60	1.58	0.40	1.86	114
3b5c*	1.50	1.75	1.66	1.78	85
3grs*	1.54	1.63	0.85	1.90	461
3sdh*	1.40	1.48	0.76	1.69	145
451c*	1.60	1.44	0.79	1.57	82
5p21*	1.35	1.75	1.38	1.92	166

Table S7 - Normalization factors for 20 different amino acids

Normalized RMSD (*nRMSD*) provides a measure of per residue side chain modelling accuracy. The residue's RMSD in the modelled structure is compared to random rotamer placement according to $nRMSD = RMSD / < RMSD >$, where $< RMSD >$ is the average RMSD of a randomly picked rotamer for each one of the 20 amino acids.

Amino acid	*<RMSD>*, Å
ALA	0.00
ARG	3.91
ASN	2.50
ASP	2.46
CYS	2.12
GLN	3.21
GLU	3.23
GLY	0.00
HIS	3.46
ILE	2.33
LEU	2.60
LYS	3.27
MET	2.96
PHE	3.94
PRO	0.73
SER	1.63
THR	1.96
TRP	4.77
TYR	4.46
VAL	1.96

Table S8 - Interface remodelling for 20 protein-protein hetero-complexes

Twenty protein complexes were remodelled with Hunter. Side chain prediction accuracy was calculated separately for all protein residues and for interface residue only. Each protein complex is identified as a 4-letter PDB ID and two 1-letter chain identifiers.

Complex	Resolution, Å	Hunter	
		All residues	Interface residues
1a4yAB	2.00	1.76	1.88
1avwAB	1.75	1.38	0.98
1cgiEI	2.30	1.58	1.67
1cmxAB	2.25	1.90	1.54
1cseEI	1.20	1.44	1.62
1cxzAB	2.20	1.97	1.79
1d09AB	2.10	1.77	2.00
1dhkAB	1.85	1.67	1.46
1dtdAB	1.65	1.50	1.81
1eayAC	2.00	1.70	2.09
1eg9AB	1.60	1.69	1.82
1emvAB	1.70	1.54	1.07
1f60AB	1.67	1.70	1.06
1finAB	2.30	1.69	1.87
1jtdAB	2.30	1.45	1.35
1lfdAB	2.10	1.95	0.92
1smpAI	2.30	1.40	1.79
1ycsAB	2.20	1.95	1.55
2kauAC	2.00	1.66	1.98
2sicEI	1.80	1.73	2.39
		1.67	1.63

Table S9 - Parameter set used in the Lennard-Jones calculation

The well depths are directly from CHARMM19 [3], while the radii are from the optimization using the 1-dimensional distributions from the PROBE run (see Methods for details).

	Atom class	Radius	Well depth
1	carbonyl carbon	1.99	0.1200
2	carboxyl carbon	1.92	0.1200
3	aliphatic carbon with 1 H	2.31	0.0486
4	aliphatic carbon with 2 H	2.12	0.1142
5	aliphatic carbon with 3 H	2.10	0.1811
6	aromatic carbon	1.95	0.1200
7	nitrogen bound to one hydrogen	1.71	0.2384
8	aromatic nitrogen with no hydrogens	1.74	0.2384
9	nitrogen bound to two hydrogens	1.73	0.2384
10	nitrogen bound to three hydrogens	2.14	0.2384
11	guanidinium nitrogen	1.76	0.2384
12	proline nitrogen	1.98	0.2384
13	hydroxyl oxygen	1.53	0.1591
14	carbonyl oxygen	1.57	0.1591
15	carboxyl oxygen	1.49	0.2100
16	sulphur	1.97	0.1600
17	backbone nitrogen	2.08	0.2384
18	backbone alpha carbon	2.20	0.0486
19	backbone carbonyl carbon	2.23	0.1400
20	backbone oxygen	1.76	0.1591

References

1. Lu M, Dousis AD, Ma J: **OPUS-PSP: an orientation-dependent statistical all-atom potential derived from side-chain packing.** *J Mol Biol* 2008, **376**:288-301.

2. Shen MY, Sali A: **Statistical potential for assessment and prediction of protein structures.** *Protein Sci* 2006, **15**:2507-2524.

3. Neria E, Fischer S, Karplus M: **Simulation of activation free energies in molecular systems.** *J Chem Phys* 1996, **105**:1902-1921.
